# REPRODUCTIVE AGING

**DOI:** 10.1093/geroni/igad104.0998

**Published:** 2023-12-21

**Authors:** Yousin Suh

## Abstract

Reproductive aging is a major health, personal and societal issue, but ovarian aging has received limited scientific attention. Ovarian aging has been known to influence diverse health outcomes in women including lifespan, cardiovascular disease, metabolic syndromes, neurodegenerative disorders and various types of cancer. Yet, the mechanisms behind this broad implication remain elusive. This session will explore recent insights into the molecular, cellular and genetic basis of ovarian aging and new intervention targets to maintain reproductive health and later health outcomes in our aging female population. Target audience: This inter-disciplinary session is designed to educate basic scientists, physician-scientists, clinicians, and a general interest audience of providers or industry representatives interested in understanding the mechanisms of ovarian aging and its sequalae.

